# Opinion of Belgian Egg Farmers on Hen Welfare and Its Relationship with Housing Type

**DOI:** 10.3390/ani6010001

**Published:** 2015-12-22

**Authors:** Lisanne M. Stadig, Bart A. Ampe, Suzy Van Gansbeke, Tom Van den Bogaert, Evelien D’Haenens, Jasper L.T. Heerkens, Frank A.M. Tuyttens

**Affiliations:** 1 Institute for Agricultural and Fisheries Research (ILVO), Animal Sciences Unit, Farm Animal Welfare and Behaviour Research Group, 9090 Melle, Belgium; bart.ampe@ilvo.vlaanderen.be (B.A.A.); evelien.dh@hotmail.com (E.D.-H.); jasperheerkens@gmail.com (J.L.T.H.); frank.tuyttens@ilvo.vlaanderen.be (F.A.M.T.); 2 Ghent University, Faculty of Veterinary Medicine, 9820 Merelbeke, Belgium; 3 Department of Agriculture and Fisheries, Koningin Maria Hendrikaplein 70 bus 101, 9000 Ghent, Belgium; suzy.vangansbeke@lv.vlaanderen.be (S.V.G.); tom.vandenbogaert@lv.vlaanderen.be (T.V.B.)

**Keywords:** egg producer, welfare, survey, laying hen, housing, conventional cage, furnished cage, colony cage, aviary, floor housing

## Abstract

**Simple Summary:**

Until 2012, laying hens in the EU were often housed in conventional cages that offered limited space and few opportunities to perform highly motivated behaviors. Conventional cages are now banned in the EU in order to improve animal welfare. In this study, egg farmers were surveyed (winter 2013–2014) to assess whether they perceived any changes in animal welfare since changing housing systems, what role hen welfare played in choosing a new housing system, and which aspects of hen welfare they find most important. The data show that the answers differ depending on which housing system the farmers currently use and whether they had used conventional cages in the past.

**Abstract:**

As of 2012, the EU has banned the use of conventional cages (CC) for laying hens, causing a shift in housing systems. This study’s aim was to gain insight into farmers’ opinions on hen health and welfare in their current housing systems. A survey was sent to 218 Belgian egg farmers, of which 127 (58.3%) responded, with 84 still active as egg farmer. Hen welfare tended to be less important in choosing the housing system for farmers with cage than with non-cage systems. Respondents currently using cage systems were more satisfied with hen health than respondents with non-cage systems. Reported mortality increased with farm size and was higher in furnished cages than in floor housing. Feather pecking, cannibalism, smothering and mortality were perceived to be higher in current housing systems than in CC, but only by respondents who shifted to non-cage systems from previously having had CC. Health- and production-related parameters were scored to be more important for hen welfare as compared to behavior-related parameters. Those without CC in the past rated factors relating to natural behavior to be more important for welfare than those with CC. This difference in opinion based on farmer backgrounds should be taken into account in future research.

## 1. Introduction

As of 2012, the EU ban on conventional cages (CC) was implemented to improve hen welfare. This ban had major effects on egg farmers because CC had been the most prevalent housing system [1]. Most egg farmers therefore had to choose an alternative housing system for their hens. 

The most common alternative housing systems in the EU are furnished cages (FC), colony cages (CO), aviaries (AV), and floor housing (FH). From a welfare perspective, FC have several advantages over the traditional CC. They are larger (750 cm^2^ *vs.* 550 cm^2^ per hen, usually housing 10–50 hens), and have extra facilities such as a nest box, at least 15 cm of perch per hen, and litter material [2]. CO are similar to FC, but they are larger and house more hens (usually 40–60), there is more space per hen (800 cm^2^) and there are at least two perches [3]. Hens are highly motivated to access a nest box [4] and to perch [5]. Litter provides a substrate for foraging and dust bathing, which are behavioral needs [6,7]. Being able to express foraging behavior is not only beneficial for the hen herself, but also for her cage mates, because it can prevent unfulfilled foraging motivation from being redirected as feather pecking [8,9]. Health, an important aspect of animal welfare, is often presumed to be better in cage than in non-cage systems [10]. This is typically reflected in lower levels of infectious diseases in cage systems compared to litter-based systems [11] and lower mortality [12,13], although some studies have not found this difference [14,15].

FH and AV are non-cage systems. These systems have the same facilities as FC and CO (perches, litter, nest boxes), but group sizes and litter area are considerably larger [16]. In FH, all hens are housed on one level, while in AV they have access to at least two levels. Hens in non-cage systems have a spacious environment that gives them the opportunity to walk and run considerable distances, fly, and escape from negative social encounters, all of which are not possible in cage systems. On the other hand, negative behaviors such as feather pecking and cannibalism are more problematic in these housing types than in cage systems, because group sizes are larger, the peckers are more difficult to identify, and such behaviors can possibly spread through the flock [11,17,18]. Smothering, where large numbers of birds group together causing birds to suffocate, is believed to occur more often in non-cage systems, although empirical evidence is lacking, and it can also occur in cage systems [19].

FH and AV can be combined with free-range (FR) access (FH + FR; AV + FR) or with a winter garden. This entails welfare benefits such as more space per animal, more environmental enrichment, and more opportunities to perform natural behaviors such as dust and sun bathing and foraging. The latter can lead to a decreased incidence of feather pecking in FR systems [20,21,22]. On the other hand, FR access also brings welfare risks with it, such as predation by foxes and birds of prey. FR systems have sometimes also been associated with higher levels of parasitic infections [23], although other studies found no differences [24,25].

The main goal of the ban on CC was to improve hen welfare, but it is not yet known if the farmers have perceived the change in that way. At present, now that several production rounds have been completed with the new housing systems, the opinion of egg farmers about the welfare of their own hens is important because of their direct experiences. This information can be used in several ways: first, for countries outside of the EU planning to implement a ban on CC or as a case-study for both EU and non-EU countries planning to implement other legislation with similar impacts on the primary sector; second, experiences from practice can be used to help to customize applied research. The main aims of this study were to get insight into egg farmers’ opinion on: (1) the importance of hen welfare and health when choosing a housing system, (2) their satisfaction with their hens’ health and welfare, (3) possible changes in the occurrence of welfare problems since converting from CC to alternative systems, and (4) which aspects of welfare they find most important. Furthermore, we have assessed if these aspects differed between farmers using cage and non-cage housing systems, and between farmers with or without experience with CC in the past.

## 2. Experimental Section

Addresses of egg farmers in Flanders (the northern region of Belgium) were provided by the Flemish Land Agency (VLM), after approval of the *Vlaamse Toezichtcommissie voor het elektronische bestuurlijke gegevensverkeer* (Flemish Monitoring Committee for electronic administrative exchange of data). The survey was sent in the winter of 2013–2014 to all 218 egg farmers who were registered at VLM in 2011 or 2012. Egg farmers are required to register at VLM if their farm either produces more than 300 kg phosphate per year or has more than 2 ha of farmland, and they can also register on voluntary basis. Therefore all Flemish farms with more than 666 chickens received the survey.

The survey consisted of three sections; the majority of the questions were about either the housing system or the welfare of the hens. Most of the data on prevalence of the different housing systems and on how farmers experienced the ban on CC have been reported in Stadig *et al.* [26]. The focus of the current paper is on the questions about hen welfare. 

In Section 1, demographic data such as age, sex, and education of the farmer were collected. Section 2 asked active farmers about the characteristics of their henhouse (e.g., flock size, litter type, number of levels (in an aviary system), and access to a FR area). It also included questions about mortality rates and slaughter age of the previous production round, since mortality rates may indicate the general health of the birds. Section 3 comprised the largest part of the survey, with detailed questions about farmers’ opinions on subjects such as the importance of hen welfare and health when choosing their current housing system, their satisfaction with regards to hen welfare and health in these systems, whether specific welfare problems had decreased or increased after the transition from CC to alternative systems, and which aspects of hen welfare were most important (based on Welfare Quality^®^ principles and criteria). The Welfare Quality^®^ protocol was designed to assess animal welfare at group level, and contains mainly animal-based measures [27]. Each of the four principles is subdivided into several criteria (Table 1). Four extra parameters designed to measure perceptions of appropriate behavior and positive emotional state were added to the list of questions (expression of nesting behavior, expression of foraging and dust bathing behavior, presence of positive emotions, and absence of negative emotions).

After receiving the survey, farmers responded by returning the completed survey by mail, filling it out online, or by phone. If no response was received 2 weeks after sending out the survey, the farmer was telephoned and asked to participate, with a maximum of five (attempted) calls per farmer.

**Table 1:** Welfare Quality^®^ principles and criteria (adapted from Welfare Quality^®^ [27]).

Principles	Criteria
Good Feeding	Absence of prolonged hunger
	Absence of prolonged thirst
Good Housing	Comfort around resting
	Thermal comfort
	Ease of movement
Good Health	Absence of injuries
	Absence of disease
	Absence of pain induced by management procedures
Appropriate Behavior	Expression of social behaviors
	Expression of other behaviors
	Good human-animal relationship
	Positive emotional state

All continuous variables (e.g., reasons and opinions) were analyzed using a linear model. Continuous variables were considered sufficiently normally distributed based on the graphical evaluation (histogram and QQ plot) of the residuals. Statistical significance was evaluated at α = 0.05. Farmer was included as a random effect in models used to test the differences between the reasons or opinions in order to correct for the multiple answers by the same farmer. In the case of pairwise comparisons between the different types of group housing, reasons or opinions, the Tukey-Kramer adjustment for multiple comparisons was used at a total significance level of 0.05. The results were analyzed using R 3.2.2 for Windows (R Foundation for Statistical Computing, Vienna, Austria). Results shown are LS means ± SE unless stated otherwise.

## 3. Results 

The response rate of the survey was 58.3%, or 127 out of 218 farmers. Of the respondents, 84 (66.1%) were still active as egg farmers. Most of the questions in the survey were at housing system level, which had the following incidences: FC: 13, CO: 14, FH: 23, FH + FR: 9, AV: 29, and AV + FR: 8. In total, this adds up to 96 responses, meaning some farmers operated more than one housing system on their farm. More specifications about the housing systems in use (e.g., group size, farm size, and age of the building) can be found in Stadig *et al.* [26].

### 3.1. Importance of Hen Health and Welfare

**Figure 1:**
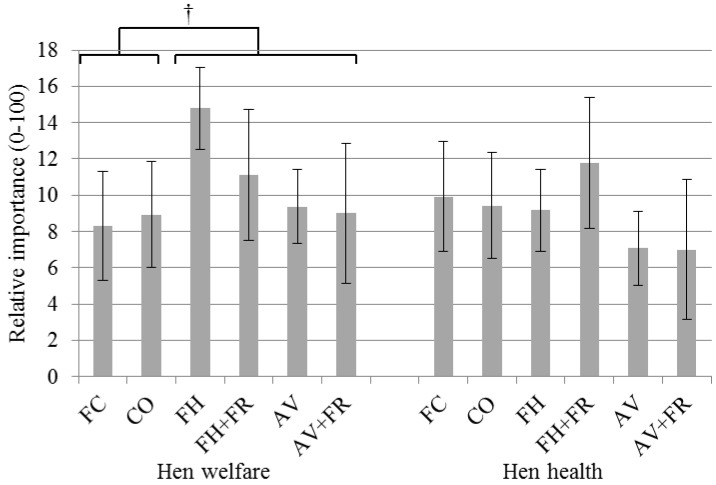
Importance of hen welfare and health as criteria for choosing a new housing system (n = 96). Respondents were asked to divide 100 points over 10 reasons, giving more points to the more important reasons. Only the results for welfare and health are shown here; for more results, see [26]. † indicates a trend towards a significant difference in importance of welfare (*p* = 0.057) between cage and non-cage systems. FC = furnished cages, CO = colony cages, FH = floor housing, FH + FR = floor housing + free-range access, AV = aviary, AV + FR = aviary + free-range access.

Farmers were asked which factors they considered most important when choosing a new housing system; they were asked to divide 100 points over 10 factors, giving more points to more important factors. Hen welfare was the third most important factor when considering which new housing system to choose; the first two were “consumer demand” and “fit within the farm”. There was no difference in the scores for hen welfare and hen health (10.6 ± 1.2 *vs.* 8.7 ± 1.2; *p* = 0.98). Hen welfare was scored as more important than operational costs and labor requirements. No clear differences were found between the different housing systems in the importance of hen health (F_5,90_ = 0.578, *p* = 0.717) and welfare (F_5,90_ = 1.922, *p* = 0.098) (Figure 1). However, the assignment of importance to hen welfare tended to differ between general categories of cage (FC and CO) and non-cage (FH, FH + FR, AV, AV + FR) systems: hen welfare in cage systems was on average scored as 2.8 ± 1.3 points less important than welfare in non-cage systems (*p* = 0.057).

### 3.2. Satisfaction with Hen Health and Welfare

**Figure 2:**
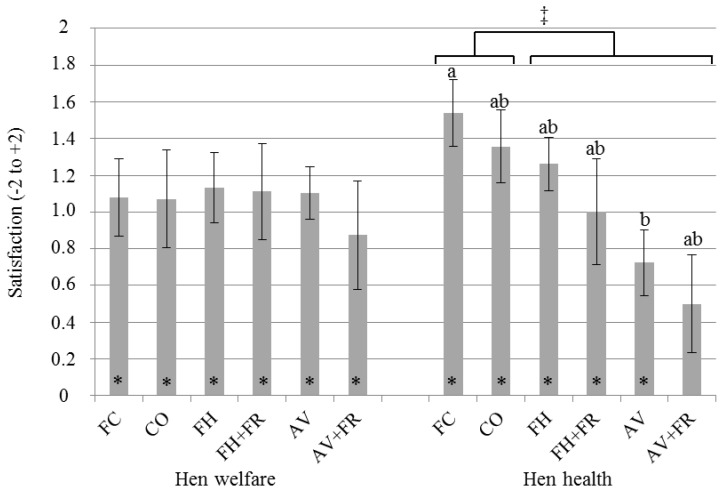
Satisfaction with hen welfare and health per housing system (n = 96). Satisfaction was scored on a scale of −2 (very unsatisfied) to +2 (very satisfied). Asterisks indicate a significant difference from the neutral score (0). Housing systems without a common superscript differ (*p* < 0.05). ‡ indicates a significant difference between cage *vs.* non-cage systems (*p* = 0.015). FC = furnished cages, CO = colony cages, FH = floor housing, FH + FR = floor housing + free-range access, AV = aviary, AV + FR = aviary + free-range access.

Satisfaction with hen health and welfare were scored on a 5-point scale from −2 (very unsatisfied) to +2 (very satisfied). In general, farmers were satisfied with hen health and welfare in their own system; all scores except for hen health in AV+FR were significantly higher than the neutral score (0; Figure 2). Regarding the satisfaction with hen welfare, there were no differences between the different housing systems. Egg farmers using FC were more satisfied with hen health than those using AV (1.54 ± 0.22 *vs.* 0.72 ± 0.15, *p* = 0.008; Figure 2), and farmers were more satisfied with hen health in cage systems than in non-cage systems (+0.48 ± 0.19, *p* = 0.015). A positive relationship between the importance of hen health in the choice for a new housing system was observed together with the satisfaction with hen health; a 1-point increase in importance equaled a 0.31-point increase in satisfaction (*p* < 0.001).

### 3.3. Mortality and Slaughter Age

**Figure 3:**
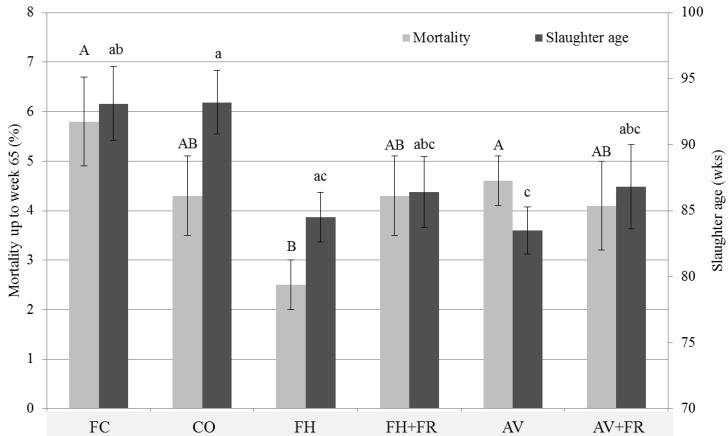
Farm-reported mortality and slaughter age per housing system. Housing systems without a common superscript differ (*p* < 0.05). FC = furnished cages, CO = colony cages, FH = floor housing, FH + FR = floor housing + free-range access, AV = aviary, AV + FR = aviary + free-range access.

Farm-reported accumulated flock mortality at 65 weeks of age of the previous production round was related to housing type (*p* = 0.006; Figure 3), with the highest mortality in FC (5.8% ± 0.9%), and lowest in FH (2.5% ± 0.5%). A relationship existed between farm-reported accumulated flock mortality and farmers’ satisfaction with hen health: when mortality increased by 1%, satisfaction decreased by 0.60 points (on a scale of –2 to +2; P = 0.012). Farm-reported mortality was also related to the number of hens on the farm (*p* = 0.023), with an increase in farm size leading to an increase in mortality. For example, a farm with 10,000 birds in an AV system would have a mean mortality of 4.25% ± 0.63% up to week 65, while for a farm with the same housing system but 30,000 hens this would be 4.64% ± 0.51%. Farm-reported mortality was not related to number of hens per housing unit or to number of finished production rounds within the current housing system. Slaughter age was higher in FC and CO (93.1 ± 2.8 and 93.2 ± 2.4 wks) than in AV (83.5 ± 1.8 wks; *p* < 0.001; Figure 3). 

### 3.4. Occurrence of Welfare Problems

**Figure 4:**
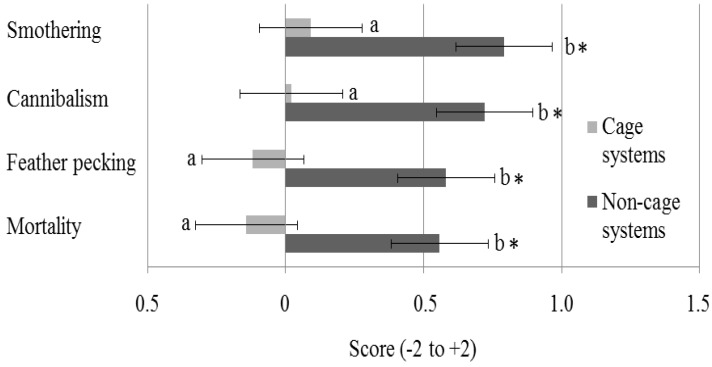
Perceived difference in the occurrence of four welfare problems in current housing systems compared to conventional cages, grouped by farmers with only cage (n = 20) or only non-cage (n = 23) systems. Lack of a common letter next to the bar indicates a difference in opinion (*p* < 0.05) between farmers currently using cage *vs.* non-cage systems. Scores ranged from –2 (much less) to +2 (much more). Asterisks indicate a significant difference from the neutral score (0).

Respondents who had used CC in the past were asked whether welfare problems had increased or decreased since the transition from those cages to alternative systems. Possible answers on a 5-point scale ranged from much less (−2) to much more (+2). In non-cage systems, smothering, cannibalism, feather pecking and mortality were perceived to have increased compared to CC, but this was not the case in cage systems (*p* = 0.004; Figure 4). 

### 3.5. Importance of Welfare Parameters

Respondents were asked about the importance of 20 welfare parameters for hen welfare, on a 5-point scale from very unimportant (–2) to very important (+2). None of the parameters were scored to be unimportant (score < 0) for hen welfare (Table 2). For the Welfare Quality® principles, respondents who had CC in the past scored good feeding as being more important than appropriate behavior (*p* = 0.007). The Welfare Quality^®^ criteria that were scored to be most important were absence of disease, absence of injuries, absence of thirst and absence of hunger, thermal comfort and comfort around nesting. Respondents without CC in the past ascribed more importance to good housing, appropriate behavior, good human-animal relationship, expression of nesting behavior, presence of positive emotions, absence of negative emotions, and expression of foraging and dust bathing behavior than respondents who used CC in the past (*p* < 0.001). No differences were reported between the scores of farmers with or without a FR housing system. It was not possible to analyze potential differences between farmers with different housing systems, because many farmers operated different housing systems on one farm.

**Table 2:** Least squares mean importance scores (on a scale from −2 to +2; very unimportant to very important) of welfare parameters according to all respondents; and presented based on whether farmers had conventional cages in the past (n = 81).

Welfare Parameter	Importance Score					
	All Respondents (n = 81)		CC in Past (n = 47)		No CC in Past (n = 34)	
Welfare Quality^®^ principles						
Good feeding		1.86 ^a^	1.77 ^ab^	1.94 ^a^		
Good health		1.83 ^ab^	1.77 ^ab^	1.88 ^a^		
Good housing		1.51 ^ab^	1.32 ^bcd^	1.74 ^ab^		*
Appropriate behavior		1.39 ^abc^	1.19 ^cde^	1.62 ^abcd^		*
Welfare Quality^®^ criteria						
Absence of disease		1.91 ^abc^	1.89 ^a^	1.884 ^a^		
Absence of injuries		1.66 ^bc^	1.60 ^abc^	1.71 ^abc^		
Absence of prolonged thirst		1.56 ^bcd^	1.55 ^abc^	1.53 ^abcde^		
Absence of prolonged hunger		1.48 ^cd^	1.47 ^abcd^	1.44 ^abcdefg^		
Thermal comfort		1.38 ^cd^	1.45 ^abcd^	1.24 ^bcdefgh^		
Comfort around resting		1.35 ^cd^	1.23 ^cd^	1.47 ^abcdef^		
Ease of movement		1.12 ^de^	1.02 ^def^	1.21 ^bcdefghi^		
Good human-animal relationship		0.92 ^ef^	0.72 ^efg^	1.15 ^cdefghi^		*
Positive emotional state		0.78 ^ef^	0.62 ^fgh^	0.95 ^efghi^		
Absence of pain induced by management procedures		0.77 ^efg^	0.72 ^efg^	0.79 ^hi^		
Expression of social behaviors		0.75 ^efg^	0.64 ^fgh^	0.85 ^ghi^		
Expression of other behaviors		0.58 ^efg^	0.49 ^gh^	0.65 ^i^		
Extra parameters						
Expression of nesting behavior		0.90 ^efg^	0.66 ^fgh^	1.18 ^bcdefghi^		*
Presence of positive emotions		0.78 ^fg^	0.53 ^fgh^	1.07 ^defghi^		*
Absence of negative emotions		0.61 ^fg^	0.36 ^gh^	0.92 ^fghi^		*
Expression of foraging and dust bathing behavior		0.50 ^g^	0.19 ^h^	0.88 ^ghi^		*

CC = conventional cages. Variables within columns without a common superscript differ (*p* < 0.05). Asterisks indicate a significant difference between respondents with and without conventional cages in the past. Non-Welfare Quality^®^ parameters are presence of positive emotions, absence of negative emotions, expression of nesting behavior, and expression of foraging and dust bathing behavior.

## 4. Discussion 

Egg farmers were asked to participate in this survey to document their opinions regarding hen health and welfare in alternative housing systems. Hen health and welfare were of average importance in choosing a new housing system. The importance of health and welfare did not differ, which can possibly be explained by farmers’ general view that health is an important part of welfare [28,29,30]. This is also reflected in the high importance attributed to ‘good health’ and ‘absence of disease’ for hen welfare. Importance of hen welfare was scored to be higher by respondents with non-cage systems than in those with cage-systems. The fact that a farmer values hen welfare more may actually be the reason why they chose a non-cage system. This is possibly related to non-cage systems giving hens more opportunities to perform natural behaviors.

Satisfaction with hen health was greater in respondents with FC than in those with AV, and was also greater in those with cage than with non-cage systems. This is not surprising, as diseases can spread more rapidly in non-cage than in cage systems [19]. This is in accordance with studies that found higher occurrences of bacterial, parasitic and viral diseases in non-cage than in cage systems [11,31]. 

This survey showed that farm-reported mortality levels, one of many indicators of hen health and welfare, up to week 65 were higher in FC than in FH. A Belgian study reported an average mortality of 4.1% at 60 weeks of age in AV systems (47 flocks), and they reasoned this relatively low percentage was possibly due to farmers’ increasing experience with AV systems, leading to improvements such as in disease control and rearing conditions [22]. This possibly indicates that mortality is not necessarily always lower in cage than in non-cage systems [14,15], which is the general belief [12,13,19,32,33,34], and which was also the perception of farmers in this survey. Another explanation for the lower farm-reported mortality levels in FH than in FC could be that dead hens can more easily go missing in non-cage systems, e.g., due to cannibalism, and are therefore not included in the mortality numbers. 

Another finding was that farm-reported mortality increased with farm size. A possible explanation could be that farmers who take care of more animals have less time to monitor each group, and consequently are more likely to miss subtle changes denoting the beginning of a problem. Larger farms may also be more likely to have flocks of different ages, which is a risk factor for disease [35].

Slaughter ages were higher in FC and CO than in AV, and numerically were also higher than in the other non-cage systems. An explanation could be that in Belgium, cage systems more often house white hens [36], which usually have a more persistent higher production performance than brown hens [15,37,38]. However, this explanation for this phenomenon could not be confirmed by this survey, because the responses did not always reveal exactly which strains of hens were used.

Feather pecking was perceived to only have increased in non-cage systems as compared to CC. This corresponds with research findings, which usually show more feather pecking in larger groups [18,39]. Cannibalism was another welfare issue that was perceived to be worse in non-cage systems, which corresponds to higher prevalence of cannibalism in non-cage systems, and/or in larger groups [11,40,41]. Cannibalism is more common in non-cage systems because it can possibly spread through the flock and is fostered by the lack of a stable social hierarchy [33,42].

Smothering was perceived to be more prevalent in the non-cage systems than in CC, which is not surprising since this problem occurs mainly in non-cage systems [19,43]. It can also occur in cage systems, but because each housing unit contains fewer animals, it is unlikely to lead to the same number of losses as in non-cage systems. It is also perhaps less likely to be recognized as smothering when it occurs in cages, which is also reflected by the perception of farmers that smothering had not increased between CC and alternative cage systems.

Regarding which welfare parameters were perceived to be most important for hen welfare, no significant differences between the four Welfare Quality® principles (good feeding, good health, good housing and appropriate behavior) were found when all respondents were grouped together. This is in contrast with findings of Tuyttens *et al.* [44] when farmers were questioned about farm animals in general. However, when considering only those respondents who had CC in the past, “ good feeding” and “ good health” were scored to be more important than appropriate behavior. Also, those farmers who did not have CC in the past scored “ appropriate behavior” to be more important than those who did have CC. This was the same for “ expression of nesting behavior” and “ expression of foraging and dust bathing behavior”(which can be classified as “ appropriate behaviors”). Of course, all alternative systems accommodate the expression of these behaviors better than CC do. Possibly, farmers who had CC in the past were more focused on production results, which is why they valued “ good feeding” and “ good health”, which are both essential for good production, more than “appropriate behavior” for animal welfare. On the other hand, farmers who did not have CC in the past were possibly more concerned with hens’ behavioral needs to begin with, which could be the reason why they did not use CC in the first place. Another explanation could be that those without CC in the past had more time to observe these behaviors as well as the hens’ motivation to express them.

When analyzing the importance attributed to the specific Welfare Quality® criteria, it appears that the “physiological” parameters (absence of disease and injuries, absence of prolonged thirst and hunger, thermal comfort) are perceived to be most important for hen welfare. Criteria more related to behavior and emotional state were scored to be less important, especially by those farmers who had CC in the past. This is in accordance with similar surveys among farmers regarding farm animals in general [44] and broiler chickens in particular [45]. The physiological parameters are also important factors for production, and a common view among farmers is that good production and health equal good welfare [28,29,30]. This is mainly the case for farmers associated with basic or top-quality assurance schemes that focus mostly on production, and less so for those associated with welfare-focused or organic schemes, which often prioritize natural behavior [46,47].

## 5. Conclusions

In conclusion, hen health and welfare were reasonably important for farmers when choosing an alternative housing system after the ban on CC. They were also quite satisfied with these aspects at the time of the survey, despite the perception that mortality, feather pecking, smothering and cannibalism had increased in non-cage systems. Further research is needed on how to remedy these problems. Good welfare still seems to be perceived to strongly correlate with good production and health, especially in respondents who had CC in the past. This study also reveals that the perception of hen welfare, health and production in alternative systems compared to in CC was influenced by the farmers’ personal experience with CC (or lack thereof) [26]. This implies that when implementing legislation such as the ban on CC, farmer background should be taken into account when informing them about, and guiding them through, transitions of this kind.
